# Alterations in Macular Microvasculature in Pterygium Patients Measured by OCT Angiography

**DOI:** 10.3390/diagnostics13091603

**Published:** 2023-04-30

**Authors:** Yingjun Cai, Zhenkai Wu, Ruolan Yuan, Pingbao Wang, Huizhuo Xu, Yi Xu, Xueyan Yao, Hua Wang, Jing Zou

**Affiliations:** 1 Eye Center of Xiangya Hospital, Hunan Key Laboratory of Ophthalmology, Central South University, Changsha 410008, China; 2 National Clinical Research Center for Geriatric Disorders, Xiangya Hospital, Central South University, Changsha 410008, China; 3 The First People’s Hospital of Changde, Changde 415000, China

**Keywords:** pterygium, optical coherence tomography angiography, macular microvascular, conjunctival microvascular

## Abstract

Previous studies have reported an association between pterygia and maculopathy, yet the underlying mechanisms and alterations to the macular microvasculature in pterygium patients have yet to be fully elucidated. Our study conducted an analysis of macular superficial vessel length density (VLD) and vessel perfusion density (VPD) to establish associations between the conjunctival and macular microvasculature in patients with unilateral and bilateral pterygia. We revealed a loss of macular microvasculature in the outer nasal (ON) region in both unilateral and bilateral pterygium patients. VLD was significantly decreased in both pterygium groups in the ON region, and VPD was notably lower in bilateral pterygium patients in the same area. Furthermore, in unilateral pterygium patients, the vessel percent pixel coverage (PPC) of the pterygium and the area of the pterygium exhibited a negative correlation with VLD in the ON region. Multiple stepwise linear regression models indicated that the PPC could best predict VLP in the ON region. Taken together, our findings suggest that patients with pterygia may be more susceptible to macular diseases, and this may be due to a compensatory increase in blood perfusion via the anterior ciliary artery. These results underscore the importance of managing maculopathy in patients with pterygia.

## 1. Introduction

A pterygium is a common ocular surface degeneration of the eye that is characterized by a fibrovascular growth towards the cornea. Previous studies have found genomic evidence in pterygium specimens for the interaction of inflammation and the subsequent immune response and fibrovascular proliferation [1,2]. The genomic alteration was pathologically validated in altered limbal epithelial cells followed by metaplastic epithelium and goblet cell hyperplasia, which induced the activation of fibroblasts, neovascularization, lymphocyte infiltration, and a remodeled extracellular matrix [3]. The development of pterygia is often accompanied by redness, irritation, dryness, tearing, and decreased vision. Compared with pseudopterygium, a pterygium is a degenerative process that often occurs on the nasal side and attaches to the cornea by invading below the epithelium and above a damaged Bowman’s membrane, causing a mild infection of the superficial stroma [4].

Pham et al. were the first to report an association between pterygia and a two- to three-fold increased risk of incident late and early age-related maculopathy [5]. Moreover, multiple studies have revealed that pterygia and macular degeneration (MD) share a common affected population; namely, individuals exposed to solar radiation (SR) [6]. Recent investigations have demonstrated a reduction in macular retinal thickness and retinal microvasculature densities in patients with pterygia [7,8]. Until now, the alterations that occur in the macula microvascular of pterygium patients as well as the underlying mechanisms of development have remained to be elucidated. 

Optical coherence tomography angiography (OCTA) is a non-invasive method to visualize retinal microvascular structures [9]. By comparing the difference between the B-scans at the same location, OCTA can reveal the movement of erythrocytes and generate a quantitative description of retinal vasculature [10]. Changes in the foveal avascular zone (FAZ) reflect alterations in the central retinal microvasculature that affect the central visual function [11]. OCTA observations in the FAZ are commonly used as indicators for the severity and progression of vascular retinopathies [11]. OCTA has been widely used in many ophthalmic diseases such as glaucoma [12] and diabetic retinopathy [13], with high repeatability and rapidity.

In this cross-sectional study, we aimed to explore the alterations in the macular microvasculature of pterygium patients and whether pterygia have an association with the incidence of MD. Our findings uncovered a novel regulatory mechanism between the conjunctive and macular microvasculature, providing new insights into the pathophysiology of maculopathy in pterygium patients.

## 2. Materials and Methods

### 2.1. Ethics

The study adhered to the guidelines of the Helsinki Declaration and was approved by the Medical Ethics Committee of Xiangya Hospital (No. 202108148). All subjects signed their informed consent.

### 2.2. Study Design and Subjects

In this cross-sectional study, pterygium patients treated from August 2021 to August 2022 in the Ophthalmology Department of Xiangya Hospital were retrospectively recruited. Considering different pathogeneses and influencing factors between unilateral and bilateral presentations of pterygia, three cohorts were constructed, which consisted of 25 eyes from 25 unilateral pterygium patients, 21 eyes randomly selected from 21 bilateral pterygium patients, and 25 eyes randomly selected from 25 age-matched healthy subjects. All patients underwent a complete ophthalmologic examination during their first visit, which included the assessment of best-corrected visual acuity (BCVA), intraocular pressure (IOP), silt lamp microscopy, and a dilated fundus examination. An OCTA examination was carried out for each patient by two experienced ophthalmologists.

Patients with confirmed primary nasal unilateral or bilateral pterygia on slit lamp microscopy were included in this study. Any patients with ocular surface disease, except primary nasal pterygia, such as blepharitis or lid structural abnormalities, conjunctival or corneal scarring, and severe dry eye were excluded. Patients with factors that could influence vessel density measurements in the macula such as severe cataracts, glaucoma, and retinal or optical nerve history disease were excluded. Patients with a diopter spherical degree >6.00–6.00 D and/or astigmatism >3.00–3.00 D, or those with a previous history of ocular surgery or trauma, or chronic topical medication over 3 months or within the past 2 weeks were excluded. Patients who used contact lenses, were allergic to mydriatics, or had systemic diseases such as hyperthyroidism, hypertension, and diabetes were also excluded from this study.

### 2.3. Anterior Segment Image Acquisition and Evaluation of the Pterygium

**Figure 1:**
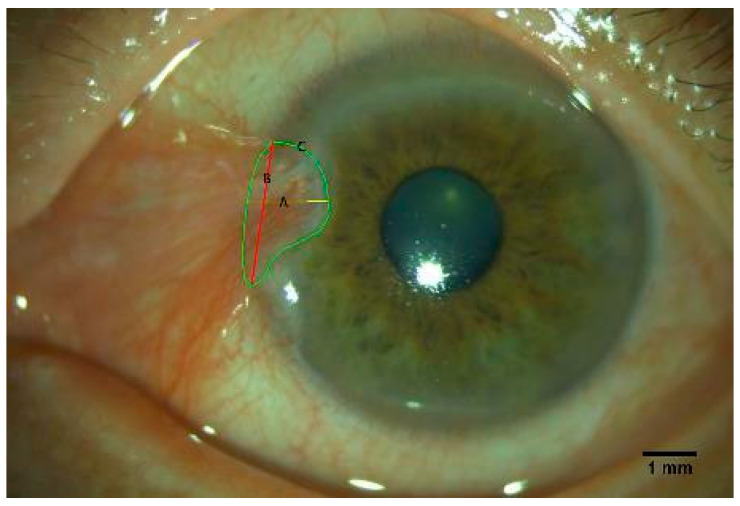
Representative photograph depicting the measurements of the pterygium parameters. (**A**) The length of the pterygium was defined as the horizontal distance from the head of the pterygium to the corneal limbus. (**B**) The pterygium width was calculated by the distance between two points where the pterygium intersected with the corneal limbus. (**C**) Area of pterygium was the pterygium-covered cornea area.

Pterygium images were acquired using a slit-lamp-mounted digital camera system (EyeSuite i9.9.3.0). Anterior segment photographs of the pterygium were taken using a 45-degree angled white beam light with a diffusion filter set at ×16 magnification. Retraction of the eyelids or the use of any topical medication was avoided during the photography to reduce reactive hyperemia. The pterygium was described by the length, width, area, and percent pixel coverage (PPC) (Figure 1). The length of the pterygium was defined as the horizontal distance from the head of the pterygium to the corneal limbus. The pterygium width was calculated as the distance between two points where the pterygium intersected with the corneal limbus. The area of the pterygium was calculated as the area of the pterygium that covered the cornea. This area measurement was manually selected and calculated with ImageJ (ImageJ 1.46; accessed on 21 March 2012; http://imagej.nih.gov/ij/; provided in the public domain by the National Institutes of Health, USA). After adding a standardized scale bar, all the parameters, including the length, width, and area of the pterygium, were measured three times to generate the respective average. 

**Figure 2:**
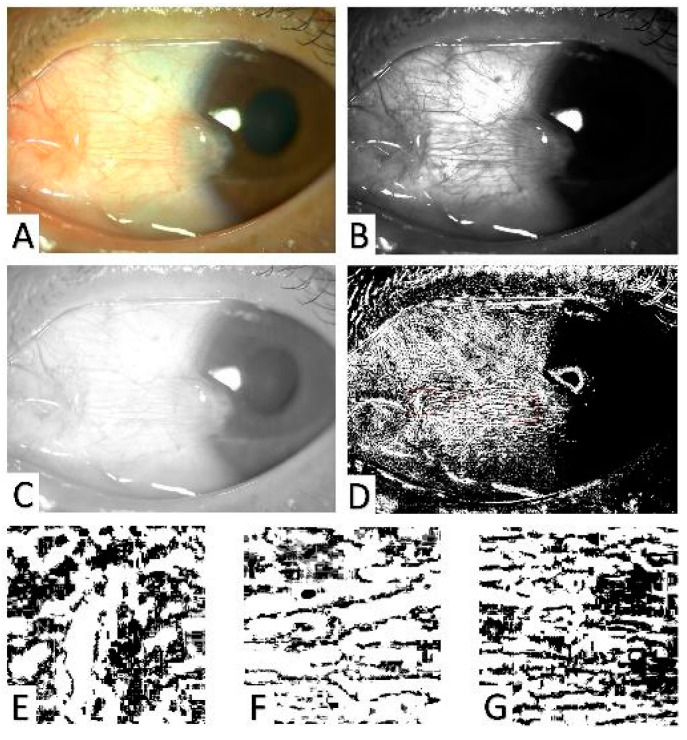
Methods for the analysis of the pterygium conjunctival vessels. (**A**) Anterior segment images of pterygium were captured using a slit-lamp-mounted camera. (**B**) The green channel was extracted from the red, green, and blue channels using ImageJ. (**C**) The sclera vessel data were extracted by enhancing the image contrast and subtracting the background. (**D**) The images were binarized using a threshold value, and three random regions of interest (ROIs) (red boxes) were selected within the pterygium. (**E**–**G**) The average of black pixel frequencies divided by the total pixel frequency for each ROI was used as the final percent pixel coverage (PPC) of the pterygium.

The pterygium conjunctival vessel perfusion density (PVP) was represented as the PPC of the pterygium vessel (Figure 2). To acquire the PPC of the pterygium, the anterior segment photographs were recorded in a JPEG format and adjusted to 3888 × 2916 pixels. The green channel offered a superior local contrast compared with the red and blue channels, making it an optimal method for visualizing the differences in intensity between vessels and the background and avoiding issues of underexposure or oversaturation [14]. To minimize background noise in the images, the green channel was split from the red, green, and blue channels using ImageJ software. The image contrast was then enhanced and the background was subtracted to extract the sclera vessel data. Next, to accurately distinguish all pixels representing the blood flow as the foreground and those that did not as the background, the images were binarized by setting the threshold grayscale value of 0 to 255. Finally, three regions of interest (ROI) of the pterygium, including the pterygium-covered cornea and conjunctiva area, were randomly selected; each ROI consisted of a square of 300 × 300 pixels [15,16]. The black pixel frequencies were then calculated for each ROI. The PPC in these ROIs was the black pixel frequencies divided by the total pixel frequency. Finally, the average of the three PPCs of each ROI was used as the final PPC of the pterygium [16,17]. 

### 2.4. Optical Coherence Tomography Angiography (OCTA) Measurements

**Figure 3:**
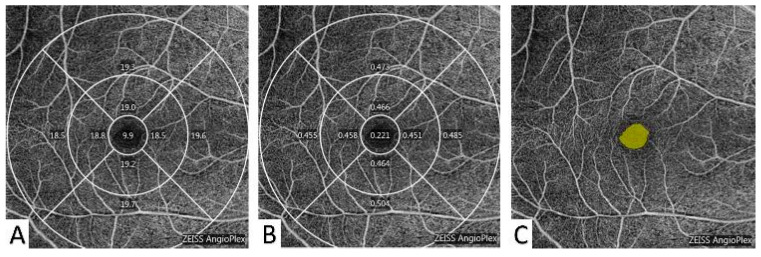
OCTA images for pterygium patients. The scanned image was divided into three annular regions according to the distance to the fovea, which were central (1 mm diameter from the fovea), inner (1–3 mm diameter from the fovea), outer (3–6 mm diameter from the fovea), and full (6 mm diameter from the fovea) regions. According to the directions, the image was separated into four quadrants, including the superior side, nasal side, inferior side, and temporal side. (**A**) The system software automatically calculated the vessel length density (VLD) and (**B**) vessel perfusion density (VPD) of the macular microvasculature. (**C**) The foveal avascular zone (FAZ) was automatically selected and calculated.

All OCTA examinations were completed using the same OCTA system (Cirrus; Zeiss; Dublin, OH, USA; software version 10.0.0.14618), which could automatically generate a 6 × 6 mm^2^ volumetric macular superficial retinal vessel image. The retinal vessel images consisted of vessels from the inner limiting membrane (ILM) to the inner plexiform layer (IPL) [18]. To collect higher-quality scans, signal strength index (SSI) values of at least 7 had to be achieved, and all patients were given short-acting dilatation 30 min before the examination. The system software automatically calculated the vessel length density (VLD) and vessel perfusion density (VPD), which were defined as the total length of the perfused vasculature and total area of the perfused vasculature in per unit area scans, respectively. The scanned image was then divided into three annular regions according to the distance to the fovea, which were central (1 mm diameter from the fovea), inner (1–3 mm diameter from the fovea), outer (3–6 mm diameter from the fovea), and full (6 mm diameter from the fovea) regions. According to the directions, the image was separated into four quadrants, including the superior side, nasal side, inferior side, and temporal side (Figure 3) [10]. Therefore, there were twelve regions in total, including the center, inner, inner superior (IS), inner nasal (IN), inner inferior (II), inner temporal (IT), outer, outer superior (OS), outer nasal (ON), outer inferior (OI), outer temporal (OT), and full. 

The OCTA system software (Cirrus; Zeiss; Dublin, OH, USA; software version 10.0.0.14618) was also applied to automatically select and calculate the FAZ indicators, including the area, perimeter, and circularity index. The FAZ circularity is the shape descriptor that can indicate the degree of FAZ similarity to a circle, and can be calculated by the following equation:

$$Circularity=\frac{4\pi \;Area}{Perimter^{2}}$$

A circularity value that is close to 0 is indicative that the FAZ shape is less circular, whereas when the value is closer to 1, it designates a perfect circle [19].

### 2.5. Statistical Analysis

All the statistical analyses were performed using SPSS (V.22.0; SPSS, Chicago, IL, USA). The Gaussian distribution of the data was tested using the Shapiro–Wilk test. Gaussian distribution data were presented as the mean ± standard deviation. Non-Gaussian distribution data were presented as a median (interquartile range). A single-factor analysis was used to compare the difference between the three groups. These analyses included the Gaussian distribution and homoscedasticity, the Kruskal–Wallis H test for the non-Gaussian distribution, or the heterogeneity of variance test. Nominal data (gender and pterygium laterality) were compared using the chi-squared test. The Bonferroni correction was used as a post hoc test and a *p <* 0.05/3 was considered to be statistically significant. Pearson’s correlation coefficient was used to test for associations between the OCTA parameters and clinical features in both the unilateral and bilateral pterygium groups. The clinical feature data were expressed as a measure of the Gaussian distribution and as Spearman correlation coefficients for the non-Gaussian distribution data. Any associations were then used to establish a regression model with a multiple stepwise linear regression to predict the clinical features of pterygia that were precited by the OCTA parameters. A *p <* 0.05 was considered to be statistically significant.

## 3. Results

### 3.1. The Clinical Features of the Participants 

A total of 71 eyes from 71 individuals were enrolled in this study, including 25 eyes from unilateral pterygium subjects, 21 eyes from bilateral pterygium subjects, and 25 eyes from healthy control subjects (Table 1). The distribution of genders, age, IOP, and SSI was similar between the three groups (*p* > 0.0167) (Table 1). Furthermore, no significant differences in the duration, laterality, length, width, area, and PPC of the pterygium were observed between the unilateral and bilateral pterygium groups (Table 1). However, the BCVA was significantly different between the three groups where, after the Bonferroni correction, the BCVA of the healthy control group was better than the unilateral pterygium group (*p* = 0.004 < 0.0167). The BCVA values between the bilateral pterygium and unilateral pterygium groups were similar (*p* = 0.045 < 0.0167) (Table 1).

### 3.2. The OCTA Parameters of the Participants

For the macular microvascular parameters, the VLD and VPD values of the ON region were significantly different in all three groups (Table 2). Compared with the controls, the VLD of patients with unilateral (*p* = 0.016) and bilateral pterygia (*p* = 0.009) was significantly decreased in the ON region. No significant difference was observed between the unilateral and bilateral pterygium groups (*p* = 0.749). The VPD of bilateral pterygium patients was significantly reduced in the ON region compared with the control group (*p* = 0.01), whereas the VPD of the unilateral pterygium group and control eyes was similar (*p* = 0.057). VLD and VPD values in the other regions were similar (*p* > 0.05). No significant differences in the FAZ indices (area, perimeter, and circularity index) were observed between the three groups (*p* > 0.05).

### 3.3. Correlation Analysis between the OCTA Parameters and the Clinical Features of Pterygia

**Figure 4:**
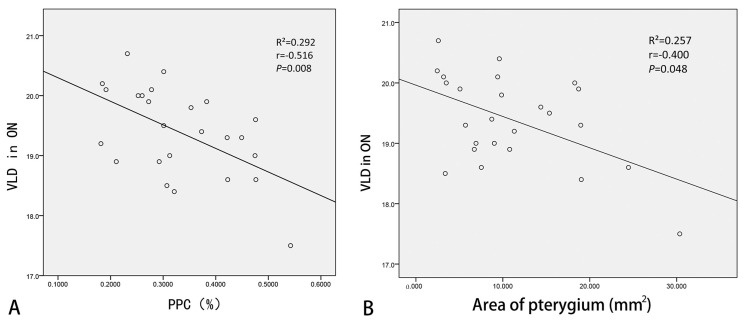
Correlation between decreased OCTA parameters and PPC (**A**) and area (**B**) of unilateral pterygium patients. PPC: percent pixel coverage; VLD: vessel length density; ON: outer nasal.

To research the influence of pterygia on decreased macular microvasculature, correction analyses were performed. Further, these analyses were used to calculate the correlation coefficient between the duration, length, width, area, and PPC of the pterygium and decreased OCTA parameters (Figure 4). In the unilateral pterygium group, the PPC and area of the pterygium were significantly negatively correlated with the VLD in the ON region (r = −0.516, *p* = 0.008; r = −0.400, *p* = 0.048). In contrast, the duration, length, width, area, and PPC of pterygia in the bilateral pterygium group were not associated with VLD and VPD in the ON region (all *p* > 0.05).

A multiple stepwise linear regression was applied to further evaluate the influence of the pterygium on macular microcirculation (Table 3). In the unilateral pterygium group, the PPC (*p* = 0.005) could best predict VLP in the ON region (adjusted R^2^ = 0.261). In the bilateral pterygium group, a multiple stepwise linear regression could not be performed as there were no associations between the pterygium-related parameters and decreased OCTA parameters.

## 4. Discussion

This is the first study to assess alterations in the macular microvasculature and the association with conjunctival microcirculation in pterygium patients. In this cross-sectional study, we recruited 25 eyes from unilateral pterygium patients, 21 eyes from bilateral pterygium patients, and 25 eyes from control subjects. After comparing the difference between these groups, we observed decreased VLD in the ON region of the unilateral pterygium group and decreased VLD and VPD in the ON region of the bilateral pterygium group. Additionally, a negative correlation between conjunctival neovascularization and macular microvasculature was noted in the unilateral pterygium group. Together, these data exhibit pterygium-related macular microvascular alterations and provide new insights into the pathophysiological mechanism of maculopathy.

In the current study, we report decreased retinal microvascularity near the ON region. This observation was confirmed by several studies. For example, Shiroma et al. [20] found that of 1645 cases of pterygia, 1583 were located on the nasal side. It was hypothesized that this may have been because of the gathering of ultraviolet radiation on the nasal corneal limbus. Coroneo et al. [21] reported that when reflected ultraviolet radiation crossed the transcameral pathway and reached the nasal limbus, the incident radiation intensity was enhanced up to 20 times. This exposure results in a continuous and fixed concentration of ultraviolet radiation on the nasal limbus, facilitating limbal stem cell damage and pterygium formation. Similarly, the occurrence of pterygia on the nasal side was also observed in patients with cortical cataracts. The Salisbury Eye Evaluation Study recruited 107 eyes to assess the location of lens opacities. The authors found that the severity of cortical cataracts in bilateral eyes was lowest in the nasal region [22]. Long-term ultraviolet radiation exposure has a cumulative effect on photic retinal injury, inducing direct DNA damage, oxidative stress, activation of inflammation, cell autophagy, and apoptosis, subsequently contributing to superficial retinal and retinal pigment epithelium degeneration [21,23]. As the macular region of the eye is a highly metabolically active area [24], we speculated that nasal macular injury may have been related to large doses of ultraviolet light that had been focused on the nasal side. This exposure then resulted in a decrease in VLD and VPD in this region.

Chanwimol et al. [25] quantified macular microvasculature density using OCTA for 22 normal eyes. The authors found that the highest macular vessel density was located on the nasal side within the nerve fiber layer. However, due to the abundant blood supply, the nasal macular microvasculature still performed a decreased phenomenon compared with the healthy controls, indicating that ultraviolet radiation plays an important role in pterygium-related maculopathy. In addition, our data indicated a negative association between the pterygium area and the PPC and VLD in the ON region of the unilateral pterygium group. Combined with previous studies, it is likely that ultraviolet radiation chronically damaged the macular microvasculature on the nasal side during the pterygium development, resulting in the decreased VLD in the ON region.

In contrast to our data, Wang et al. [7] enrolled 18 left eyes from 18 female pterygium patients and 18 eyes from 18 female healthy controls. Using OCTA examinations, the authors observed decreased superficial microvascular density, mainly on the bitemporal side. The authors hypothesized that the reduction was caused by the pterygium blocking light from entering the cornea, resulting in a reduction in retinal activity and metabolic demands. It is important to note that most of the recruited pterygium patients in Wang et al.’s study presented with severe pterygia. In their study, the average length of the pterygia was 6.25 ± 1.65 mm; the included pterygia had also crossed the pupil in the majority of the cases, resulting in significantly reduced intraocular light transmission. In contrast, the pterygium patients discussed in the current study presented with normal to mildly severe pterygia. The average length of the pterygia was 3.50 ± 1.34 mm in the unilateral cases and 2.81 ± 0.89 mm in the bilateral cases. In both groups, the pterygium had either not reached or had just reached the pupillary margin. Hence, the decreased macular vessel density on the nasal side observed in this study may have been an indicator of an early-stage macular microvasculature change in the pterygium patients. Although form deprivation induced by pterygia may be an influencing factor of macular microvascularity, in the current study, the pterygia did not restrict intraocular light transmission. Therefore, any alterations to the macular microvasculature were independent of the influence of light sheltering in our study and our data may better reflect the pathophysiological changes in pterygium patients.

Additionally, a negative association was observed between the PPC and macular microvascularity. This association was further validated in a multiple stepwise linear regression model, suggesting a potential interaction between conjunctival and macular microvasculatures. A pterygium is a degenerative change where the underlying fibrovascular tissue tends to exhibit a chronic inflammatory phenotype, and is characterized by abundant angiogenesis [26]. Angiogenesis is the process by which new vessels are formed from pre-existing vasculature, facilitated by the activation of angiogenic factors and extracellular matrix components, resulting in the formation of a new layer of pathological pterygium stroma between the corneal epithelium and Bowman’s membrane [27]. The blood supply of a pterygium can be divided into the innermost head and body, and is one of the establishing features of pterygia. The innermost head of the pterygium originates from the anterior branches of the episcleral vessel with the migration of the conjunctival epithelium; the subsequent blood supply originates from the anterior conjunctival vessels. Therefore, the head of a pterygium has a dual blood supply, originating from the conjunctival and episcleral vessels. The body of the pterygium is mainly fed by the anterior conjunctival vessel [28]. The ophthalmic artery is the first branch of the internal carotid artery. Anterior ciliary arteries branch from the muscular arteries and mainly supply the conjunctival vessels. The first branch of the ophthalmic artery–central retinal artery mainly provides blood to the inner layer of the retina [29]. 

Recently, Shi et al. [30] completed an OCTA examination study in systemic lupus erythematosus (SLE) patients, where the authors acquired macular and conjunctival microvascular images. Using these data, the authors found that the conjunctival vascular density was negatively correlated with the superficial total microvascularity in SLE patients. They speculated that the correction may have been caused by a compensatory increase in blood perfusion by the anterior ciliary artery. This finding was similar to our results, and we speculated that increased neovascularization of the conjunctival vessels supplied by the anterior ciliary artery in the pterygium may have facilitated a compensatory decrease in macular microvascular blood flow by the central retinal artery. However, apart from pterygium formation, there are many factors that may affect macular microvasculature such as age [31], sex [32], smoking [32], body composition [33], and diet [34]. Hence, pterygium formation may be a potential factor related to macular microvasculature, and further experimental research is needed to verify the speculation on a pathophysiological level.

This study had several limitations. First, whilst 25 eyes for unilateral pterygium subjects, 21 eyes for bilateral pterygium subjects, and 25 eyes for healthy control subjects were retrospectively selected for this study, most patients presented with a normal to mild degree of pterygium. Future studies should focus on enrolling a larger number of patients with varying degrees of disease severity to better research altered macular microvasculature-related pterygia. Second, VLD and VPD were only calculated for the superior macular vessel. Further research should examine the deep macular and choroidal vessels to obtain a more holistic observation of pterygium pathogenesis. Third, these patients were only measured at the first visit. A long-term follow-up would allow for a better assessment of pterygium development as well as the ability to assess macular microvasculature effects. Additionally, the ocular microbiome plays an essential role in the development of ocular disease. Extensive scientific investigations have substantiated the essential role of the gut microbiome (GM) in the pathogenesis of age-related macular degeneration (AMD) by modulating critical processes such as angiogenesis, scavenger and cytokine receptor activity, and the inflammatory response [35]. Moreover, the ocular surface microbiome has shown a connection to the gut microbiome [36]. Thus, we speculated that the GM and ocular microbiome may jointly modulate pterygium-associated maculopathy. Metagenomics could be utilized as a novel tool for the detection of the ocular surface microbiome, revealing the underlying molecular mechanisms. In future research, we may gain a deeper understanding of the mechanisms underlying the pterygium and its associated maculopathy through the study of the microbiome.

## 5. Conclusions

Our research presented an association between conjunctival vascularization and macular microvasculature in pterygium patients, indicating that patients with pterygia may be more likely to suffer from macular diseases. Our results suggest the need to strengthen maculopathy management in pterygium patients.

## Figures and Tables

**Table 1:** Clinical features and ophthalmologic characteristics of study participants.

Characteristic	Unilateral Pterygium (*n* = 25)	Bilateral Pterygium (*n* = 21)	Control (*n* = 25)	*p*-Value
Age, years	56.00 (50.5–59.5)	55.6 ± 7.4	51.3 ± 10.7	0.215
Gender no. (%)				1.000
Female	12 (48.0)	10 (47.6)	12 (48.0)	
Male	13 (52.0)	11 (52.4)	13 (52.0)	
BCVA, logMAR	5.0 (5.0–5.1)	5.0 (5.0–5.1)	5.1 (5.1–5.1)	0.006 ^a^
IOP, mmHg	14.6 ± 2.0	15.5 ± 2.3	14.4 ± 2.2	0.235
Eye laterality no. (%)				0.781
Right	13 (52.0)	9 (42.9)	13 (52.0)	
Left	12 (48.0)	12 (57.1)	12 (48.0)	
Duration time, years	3.0 (1.0–8.5)	3.0 (0.4–8.0)	/	0.646
Pterygium width, mm	5.5 ± 1.6	4.7 ± 1.2	/	0.066
Pterygium length, mm	3.5 ± 1.3	2.8 ± 0.9	/	0.053
Pterygium area, mm^2^	11.0 (5.4–16.8)	7.0 ± 3.8	/	0.066
PPC, %	33.1 ± 10.3	30.8 ± 14.0	/	0.848
SSI	10.0 (8.0–10.0)	10.0 (8.5–10.0)	9.0 (9.0–10.0)	0.975

The Gaussian distribution data were presented as mean ± standard deviation; the non-Gaussian distribution data were presented as median (interquartile range). Single-factor analysis was used to make comparisons between the three groups for Gaussian distribution data. The Kruskal–Wallis H test was used for data that did not fit a non-Gaussian distribution. Bonferroni correction was used for the post hoc test. BCVA: best-corrected visual acuity; IOP: intraocular pressure; PPC: percent pixel coverage; SSI: signal strength index. ^a^: significant difference between the unilateral pterygium group and the control group.

**Table 2:** OCTA parameters of the participants.

OCTA Parameters	Unilateral Pterygium	Bilateral Pterygium	Control	*p*-Value
VLD				
Central	6.82 ± 3.54	7.18 ± 3.38	7.76 ± 2.69	0.359
Inner	17.10 (14.70–18.55)	17.00 (14.78–18.48)	17.56 ± 1.65	0.171
IS	16.50 (14.05–18.75)	17.10 (15.30–18.68)	17.57 ± 1.92	0.173
IN	16.80 (13.80–18.80)	17.85 (16.40–18.30)	18.45 (17.10–19.23)	0.050
II	17.20 (14.65–18.75)	16.45 (14.03–18.65)	17.63 ± 1.45	0.097
IT	17.30 (15.15–18.65)	17.00 (14.43–18.25)	17.95 (16.23–18.88)	0.350
Outer	17.31 ± 1.81	17.55 (15.90–18.85)	17.76 ± 1.51	0.497
OS	18.00 (16.70–19.00)	17.95 (15.45–18.80)	17.70 ± 1.46	0.615
ON	19.20 (18.75–20.00)	19.24 ± 0.73	19.95 (19.53–20.48)	0.013 ^a^
OI	17.34 ± 2.01	16.50 (15.3–17.7)	17.95 ± 1.40	0.108
OT	17.10 (13.55–17.80)	15.51 ± 3.17	16.25 (13.93–18.45)	0.672
Full	16.80 ± 2.05	17.10 (15.50–18.60)	17.44 ± 1.48	0.423
FAZ				
Area	0.27 (0.15–0.38)	0.30 ± 0.13	0.29 ± 0.10	0.789
Perimeter	2.13 ± 0.62	2.23 ± 0.51	2.33 ± 0.46	0.405
Circularity	0.73 (0.64–0.79)	0.75 (0.63–0.79)	0.67 ± 0.13	0.514
VPD				
Central	0.15 ± 0.08	0.16 ± 0.08	0.17 ± 0.06	0.266
Inner	0.40 (0.34–0.45)	0.41 (0.36–0.44)	0.42 ± 0.04	0.215
IS	0.38 (0.33–0.45)	0.42 (0.36–0.45)	0.42 ± 0.05	0.267
IN	0.40 (0.33–0.44)	0.43 (0.39–0.43)	0.44 (0.40–0.45)	0.052
II	0.40 (0.34–0.45)	0.39 (0.33–0.44)	0.42 ± 0.04	0.089
IT	0.41 (0.35–0.44)	0.40 (0.35–0.44)	0.41 ± 0.06	0.516
Outer	0.43 ± 0.05	0.43 (0.39–0.46)	0.43 (0.42–0.47)	0.492
OS	0.44 (0.41–0.47)	0.45 (0.39–0.46)	0.44 ± 0.04	0.787
ON	0.48 (0.46–0.49)	0.47 ± 0.02	0.49 (0.48–0.50)	0.027 ^b^
OI	0.44 ± 0.05	0.42 (0.39–0.47)	0.44 ± 0.04	0.392
OT	0.41 (0.30–0.44)	0.41 (0.31–0.44)	0.39 (0.34–0.46)	0.714
Full	0.41 ± 0.05	0.42 (0.38–0.45)	0.42 (0.41–0.46)	0.400

The Gaussian distribution data were presented as mean ± standard deviation; the non-Gaussian distribution data were presented as median (interquartile range). Single-factor analysis was used to compare the differences between the three groups, which were expressed as Gaussian distribution and homoscedasticity, the Kruskal–Wallis H test for non-Gaussian distribution, or heterogeneity of variance. Bonferroni correction was used for the post hoc test. VLD: vessel length density; VPD: vessel perfusion density; FAZ: foveal avascular zone; IS: inner superior; IN: inner nasal; II: inner inferior; IT: inner temporal; OS: outer superior; ON: outer nasal; OI: outer inferior; OT: outer temporal. ^a^ Significant difference between the unilateral pterygium group and the control group, and between the bilateral pterygium group and the control group. ^b^ Significant difference between the bilateral pterygium group and the control group.

**Table 3:** Predictors of VLD by multiple linear regression analysis in patients with unilateral pterygia.

Group	Parameter	Regression Formula	r	Adjusted R2	Durbin–Watson	*p*-Value
Unilateral pterygium	VLD in ON	20.691–0.541 PPC	0.541	0.261	2.122	0.005

VLD: vessel length density; PPC: percent pixel coverage; ON: outer nasal; r: correlation coefficient; R^2^: coefficient of determination.

## Data Availability

The data are unavailable due to privacy or ethical restrictions.
